# Hopeless, Offensive, and Incurable

**Published:** 1887-02-12

**Authors:** Emilia Vincent

**Affiliations:** Lady in Charge. Nursing Home, Reading


### Difficult Cases.

[Inquiries and Cases are always welcome for this Column.]

### Hopeless, Offensive, and Incurable.

I am glad to see from time to time some of the many sad cases of incurable suffering that exist, brought to light in the pages of The Hospital under the head of "Difficult Cases." I am afraid the old proverb, "out of sight out of mind," has partly been the cause why homes for these poor unseen sufferers have, until lately, taken but a small share among the many institutions existing in our country. I am glad now to hear frequently of fresh Homes for Incurables springing up in various parts of England, yet many more are needed, particularly some for the very bad cases of incurable illness, or, in plain English, the cases that are unpleasant, and very trying to nurse. In a very small way an attempt has been made to meet this want in Reading. The Nursing Home, Brownlow Road, was opened in December 1878. Since then, thirty sufferers have been committed to our care. Of these, twelve have died in the Home, eight are now with us, and ten have left. Of those who died, six were cases of cancer, one of lupus. Of those now with us, four are so utterly helpless that they cannot be moved without the help of two, sometimes three people; and another is a sad case of internal ulceration. It will not surprise those who know something of illness to hear that these very helpless, and often dying inmates, are more expensive to nurse than those suffering from acute illness. Often they cannot even turn in bed, or feed themselves, without help, so that much time has to be given them, and a larger staff of attendants is required than for those who can in any way assist themselves. When vacancies occur we generally select the most urgent case on our list.

Hitherto, two of the three small houses used for the Home have been lent to us. It is uncertain how long we can reckon on this help; we want, therefore, to raise £3,000 to purchase the whole property, and place the Institution on a more permanent footing. Towards this we have at present only £300. The two houses lent have been thrown into one, and fitted with conveniences for nursing; but the third house cannot be so well adapted unless the property is purchased, and the owner is willing to sell when the purchase-fund is complete. We have many kind friends in Reading who have helped us to carry on our work for eight years, but as our patients come from all parts of England, we hope to receive contributions to our purchase-fund from all parts also. I shall be glad to send a little sketch I last year drew up of the work done in the Home to any who would like to know more about us. Canon Garry is our vicar and chaplain, and O. C. Maurice, Esq., is our hon. medical attendant. The hon. treasurer, D. M. Gardner Esq., Lindeth, Bath Road, Reading, will thankfully receive any donations, or they can be paid to the bankers, Stephens, Blandy and Co., Market Place.

Emilia Vincent,
Lady in Charge.

Nursing Home, Reading.

